# Effect of AI-powered patient management systems in dental clinics

**DOI:** 10.6026/973206300222045

**Published:** 2026-04-30

**Authors:** S Mohammed Harris, Revanth S Soonthodu, Saloni Bharti, Maneesha Das, Christina Sarah James, K Aadisha

**Affiliations:** 1 Department of Periodontics, SRM Dental College, Bharathi Salai, Ramapuram, Chennai-89, Tamil Nadu, India; 2 Department of Orthodontics, KVG Dental College and Hospital, Kurunjibhag, Sullia, Karnataka 574327, India; 3 Department of Dentistry, ESIC Medical College and Hospital, Kateshar, Bihta, Bihar 801103, India; 4 Department of Conservative Dentistry and Endodontics, Institute of Dental Sciences, Siksha 'O' Anusandhan University, Bhubaneswar-751003, Odisha, India; 5 Department of Oral Pathology, Travancore Dental College, Thattamala, Mylapure, Palathara, Kollam, Thazhuthala, Kerala 691020, India; 6 Department of Public Health, Sri Ramachandra Institute of Higher Education and Research (Deemed to be University) No. 1, Ramachandra Nagar, Porur, Chennai - 600116, Tamil Nadu, India

**Keywords:** Artificial intelligence (AI), patient management system, dental clinics, healthcare efficiency, clinical decision support

## Abstract

Dental clinics frequently experience operational inefficiencies due to fragmented data systems and manual administrative processes. Therefore, it is of interest to evaluate the impact of AI-powered patient management systems on workflow efficiency, data accuracy and patient satisfaction across five clinics. Appointment scheduling time reduced from 9.6 ± 2.4 to 3.2 ± 1.1 minutes, billing errors declined from 12.5 ± 3.8% to 3.7 ± 1.9% and data entry accuracy improved to 96.2 ± 2.7%. Patient satisfaction scores increased across communication, access to records, waiting time experience and overall service quality. Thus, AI integration was associated with measurable improvements in administrative precision and clinical workflow performance.

## Background:

Efficient administrative management is fundamental to the functional stability of modern dental clinics. Inadequate scheduling systems, fragmented electronic records and manual billing procedures increase operational delays and administrative errors [1]. These inefficiencies negatively influence workflow coordination, resource utilization and patient satisfaction. Moreover, inconsistent documentation and delayed data retrieval may compromise clinical decision-making and continuity of care [2]. Artificial intelligence has increasingly been integrated into healthcare information systems to enhance automation, predictive analytics and data management [3]. AI-driven platforms employ machine learning algorithms to optimize appointment allocation, reduce missed visits and automate billing verification [4]. Such systems also support structured electronic health records that improve data integrity and accessibility [5]. In dentistry, AI applications have predominantly focused on diagnostic imaging, radiographic interpretation and treatment planning. However, the operational implications of AI-powered patient management systems remain comparatively underexplored [6]. AI-enabled administrative systems can potentially reduce clerical workload, minimize transcription errors and standardize communication protocols. Automated reminders and real-time data synchronization may improve patient adherence and clinic throughput [7]. Predictive analytics may further assist in anticipating patient flow and resource demands. Despite increasing adoption of AI management platforms, quantitative evidence evaluating measurable improvements in efficiency, accuracy and patient satisfaction within dental clinics remains limited [8]. Therefore, it is of interest to study the impact of AI-powered patient management systems on workflow efficiency, data accuracy and patient satisfaction in dental clinics.

## Materials and Materials:

This descriptive and analytical study evaluated the operational impact of AI-powered patient management systems in dental clinics. Five dental clinics that had implemented AI-driven management platforms for at least six months were purposively selected. The inclusion criterion ensured adequate exposure to system functionality. The study population comprised 25 dental professionals, 10 administrative staff members and 50 patients. Primary data were collected using structured questionnaires and brief semi-structured interviews. Questionnaires assessed workflow efficiency, data accuracy, and communication quality and satisfaction metrics. Patient responses were recorded using a 5-point Likert scale (1 = very poor, 5 = excellent). Operational parameters evaluated included appointment scheduling time, billing error rate, data entry accuracy and time saved per patient. Pre-implementation values were obtained from clinic records and staff reports. Post-implementation metrics were collected after sustained AI system usage. Secondary data were obtained from peer-reviewed literature to contextualize findings. Three commonly used AI-based management platforms were examined, including Denticon, CareStack and Tab32. Functional domains analyzed included automated scheduling, electronic health record integration, billing automation and predictive analytics. Comparative assessment focused on workflow streamlining, error reduction and decision-support capabilities. Quantitative data were analyzed using descriptive statistics and percentage change calculations. Qualitative responses were thematically categorized to identify recurring perceptions regarding workload reduction, communication enhancement and clinical support. Ethical standards were maintained throughout the study. Participants were informed of the study objectives and provided consent prior to participation. Confidentiality of institutional and patient data was strictly preserved.

## Results:

Five dental clinics utilizing AI-powered patient management systems were included in the analysis. A total of 85 participants were evaluated, comprising 25 dental professionals, 10 administrative staff members and 50 patients. The mean duration of AI system usage was 9.4 ± 2.1 months. The average daily patient flow across clinics was 38.2 ± 6.4 patients. The mean staff strength per clinic was 12 ± 3. Appointment scheduling time decreased from 9.6 ± 2.4 minutes before AI implementation to 3.2 ± 1.1 minutes after implementation, representing a 66.7% reduction. Billing error rates declined from 12.5 ± 3.8% to 3.7 ± 1.9%, corresponding to a 70.4% reduction. Data entry accuracy improved from 78.6 ± 5.3% to 96.2 ± 2.7% following AI integration. The mean time saved per patient after AI adoption was 6.5 ± 1.8 minutes. Patient satisfaction with appointment reminders increased from 2.8 ± 0.6 to 4.6 ± 0.5 on a 5-point scale. Ease of access to dental records improved from 3.1 ± 0.8 to 4.7 ± 0.4. Waiting time experience scores increased from 2.9 ± 0.7 to 4.4 ± 0.5. Overall patient satisfaction raised from 3.0 ± 0.6 to 4.8 ± 0.3. Among dental professionals, 84% agreed that AI improved clinical decision-making, 88% agreed that it reduced administrative workload, 92% agreed that it enhanced communication, 90% agreed that it improved time management and 85% agreed that it increased patient retention and trust. Table 1 (see PDF) shows five clinics with a total of 85 participants, a mean AI usage duration of 9.4 ± 2.1 months, an average daily patient flow of 38.2 ± 6.4 and means staff strength of 12 ± 3. Table 2 (see PDF) compares workflow efficiency before and after AI implementation and shows a reduction in appointment scheduling time from 9.6 ± 2.4 to 3.2 ± 1.1 minutes and billing error rate from 12.5 ± 3.8% to 3.7 ± 1.9%, while data entry accuracy increased from 78.6 ± 5.3% to 96.2 ± 2.7% and time saved per patient averaged 6.5 ± 1.8 minutes. Table 3 (see PDF) demonstrates improvement in patient satisfaction scores, with appointment reminder satisfaction increasing from 2.8 ± 0.6 to 4.6 ± 0.5, ease of access to records from 3.1 ± 0.8 to 4.7 ± 0.4, waiting time experience from 2.9 ± 0.7 to 4.4 ± 0.5 and overall satisfaction from 3.0 ± 0.6 to 4.8 ± 0.3. Table 4 (see PDF) highlights professional perceptions showing agreement rates of 84% for improved clinical decision-making, 88% for reduced administrative workload, 92% for enhanced communication, 90% for improved time management and 85% for increased patient retention and trust.

## Discussion:

This study evaluated the operational and perceptual impact of AI-powered patient management systems in dental clinics using quantitative workflow metrics and stakeholder feedback. The findings demonstrated substantial reductions in appointment scheduling time and billing errors following AI integration [9]. Data entry accuracy improved markedly, indicating enhanced record precision and system reliability. Time saved per patient further reflected measurable gains in operational efficiency. These objective improvements were accompanied by significant increases in patient satisfaction across communication, record accessibility, waiting time experience and overall service quality [10]. The reduction in scheduling time suggests that algorithm- based appointment allocation improves resource utilization and minimizes administrative delays. Automated billing verification likely contributed to the observed decline in error rates. Enhanced data accuracy indicates reduced manual transcription and improved digital synchronization of clinical records [11]. Such improvements directly influence workflow stability and reduce administrative burden.

High agreement rates among dental professionals regarding workload reduction and workflow enhancement support the quantitative findings. Patient-reported outcomes revealed substantial improvements in satisfaction domains linked to communication and access to information. Automated reminders and real-time record access likely improved transparency and continuity of care. Reduced waiting time perception suggests more efficient patient flow coordination. These findings demonstrate that AI integration affects not only administrative metrics but also patient-centered service dimensions [12]. Importantly, this study integrates operational indicators, patient-reported outcomes and professional perceptions within a single evaluative framework. Previous investigations often focused either on diagnostic AI applications or theoretical workflow benefits without measurable clinic-level data [13]. By quantifying reductions in scheduling time, billing errors and documenting improvements in satisfaction scores, this study provides empirical evidence linking AI implementation to tangible efficiency outcomes in routine dental practice [14]. The inclusion of both administrative and experiential parameters strengthens the internal validity of the assessment and clarifies the multidimensional impact of AI systems [15]. Despite these findings, several considerations remain. The sample size was limited to five clinics and implementation environments may vary across institutions. The duration of AI usage averaged less than one year, which may not capture long-term sustainability or cost-effectiveness. Differences among software platforms were not subjected to comparative statistical modeling. Data privacy, cybersecurity and infrastructure costs also require ongoing evaluation as digital systems expand. Overall, the results demonstrate that AI-powered patient management systems are associated with measurable improvements in administrative efficiency, data precision and patient satisfaction within dental clinics. The integration of quantitative workflow metrics with stakeholder perception analysis provides a structured evaluation model that may inform future digital health implementation strategies in dentistry.

## Conclusion:

AI-powered patient management systems were associated with significant reductions in administrative errors, improved workflow efficiency and enhanced patient satisfaction in dental clinics. The integration of automated scheduling, accurate digital records and structured communication tools demonstrated measurable operational benefits at the clinic level. Thus, strategic implementation of AI-based management platforms can strengthen efficiency, precision and patient-centered service delivery in contemporary dental practice.
